# Reflection and Refraction of the L‐O Mode 5 kHz Saturn Narrowband Emission by the Magnetosheath

**DOI:** 10.1029/2021GL096990

**Published:** 2022-03-14

**Authors:** S. Y. Wu, S. Y. Ye, G. Fischer, C. M. Jackman, J. Wang, J. D. Menietti, B. Cecconi, M. Y. Long

**Affiliations:** ^1^ Department of Earth and Space Sciences Southern University of Science and Technology Shenzhen People's Republic of China; ^2^ Space Research Institute Austrian Academy of Sciences Graz Austria; ^3^ School of Cosmic Physics Dublin Institute for Advanced Studies Dublin Ireland; ^4^ Department of Physics and Astronomy University of Iowa Iowa City IA USA; ^5^ LESIA, Observatoire de Paris Université PSL CNRS Sorbonne Université Université de Paris Meudon Paris France; ^6^ Department of Space Physics School of Electronic Information Wuhan University Wuhan People's Republic of China

## Abstract

The reflection‐by‐sheath mechanism of 5 kHz narrowband emissions (NB) at Saturn is confirmed by Cassini observations during several crossings of the magnetopause, which show that the 5 kHz NB can be prevented from escaping Saturn's magnetosphere. The L‐O mode 5 kHz NB remained visible in areas of low plasma density but disappeared in regions of high plasma density. In three cases, NB disappeared immediately after the crossings of Saturn's magnetopause. A possible reflected NB event observed near the magnetosheath is discussed. This mechanism can help explain the 5 kHz NB observed at low latitudes outside the Enceladus plasma torus and their upper frequency limit variations. This mechanism significantly improves the current understanding of the 5 kHz NB.

## Key Points

- The 5‐kHz narrowband emissions (NB) from Saturn can be reflected by density structures in the magnetosheath
- Reflection leads to depolarization and trapping of NB inside Saturn's magnetosphere
- The upper frequency limit of trapped NB depends on variations in the magnetosheath and the solar wind

## 1 Introduction

Saturn narrowband emissions (NB) were discovered by the Voyager Plasma Wave System (Gurnett et al., 1981; Scarf & Gurnett, 1977) and usually appear as Z‐mode or L‐O mode emissions near both 5 and 20 kHz (Gu et al., 2013; Ye, Menietti, et al., 2010). Emissions near 10 kHz are also reported (Menietti et al., 2009), but this needs further study. Case studies have shown that the Z‐mode NB (at both 5 and 20 kHz) can be generated by temperature anisotropy and loss cone distributions in the low latitude region interior to the plasma torus, whereas the modulation analysis and direction finding results indicate a possible auroral source (Menietti et al., 2011, 2016, 2018; Ye, Gurnett, et al., 2010). More events to check the plasma distribution near the source region will help to further explore the generation of the Z mode NB. According to the Z‐mode dispersion relation, the initially generated Z‐mode NB would be trapped in a "Channel" formed by the iso‐surfaces of the *f*_uh_ and *f*_L = 0_ (*f*_uh_ upper hybrid frequency, *f*_L = 0_ Z‐mode cut‐off frequency; Sonwalkar et al., 2004). These “trapped” Z‐mode NB can mode convert to the “free space” L‐O mode NB at density gradients (Menietti et al., 2019; Ye, Menietti, et al., 2010). Due to the blockage of the high density plasma torus (centered at the equatorial plane) generated by the cryovolcanic activity of the moon Enceladus, the L‐O mode NB originally generated interior to the plasma torus would propagate to high latitudes unhindered, creating a low latitude shadow zone outside the plasma torus (S. Wu et al., 2021; Ye, Menietti, et al., 2010). However, 5 kHz NB is still observed at low latitudes outside the plasma torus. How did these NB bypass the plasma torus?

S. Wu et al. (2021) proposed that Saturn's magnetosheath may reflect the lowest frequencies of L‐O mode NB, as electron density measurements in Saturn's magnetosheath (Sergis et al., 2013) sometimes went to above 0.3 cm^−3^, which is high enough to reflect the 5 kHz NB. However, that work only covered the statistical characteristics of 5 and 20 kHz NB and did not provide evidence on the reflection mechanism.

## 2 Distribution of the 5 kHz NB Emissions

**Figure 1:**
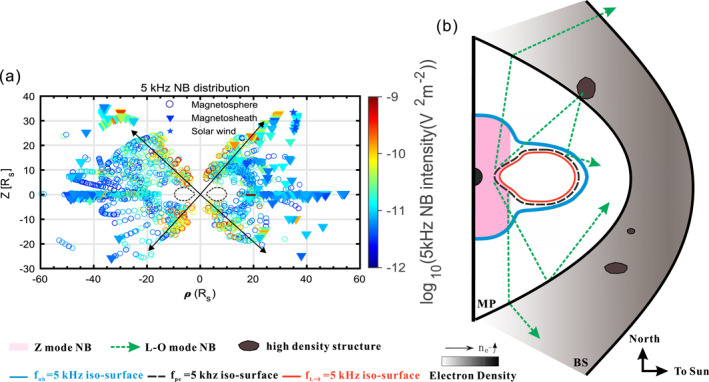
Panel (a), the observed 5 kHz NB distribution in the ρ‐Z plane (ρ: horizontal distance to the spin axis, $\sqrt{x^{2}+y^{2}}$, positive for the dayside, negative for the nightside). The black dashed circle marks the rough location of the plasma torus. The markers in circle, triangle, and star represent the cases observed in the magnetosphere, magnetosheath, and solar wind, respectively. Color codes are the 5 kHz NB intensities obtained by integrating the mean spectral density during the event time and between 3 and 8 kHz. The black arrows indicate the beaming to high latitudes of 5 kHz NB and inhibition of their propagation through the Enceladus plasma torus. Panel (b), Cartoon illustration of the Z mode trapping region and the reflection‐by‐sheath mechanism for 5 kHz NB. The blue and red lines (iso‐surfaces of *f*_uh_ and *f*_L = 0_) depict the theoretical Z‐mode trapping region (Ye, Menietti, et al., 2010). The pink region marks the location where the 5 kHz Z‐mode NB was observed in the statistical study of S. Wu et al. (2021). The dashed black circular line represents a rough picture of the cut‐off frequency of the 5 kHz NB. The green arrows show examples of the 5 kHz L‐O mode NB propagation path. Two lines of "MP" and "BS" represent the magnetopause and the bow shock.

We used two key catalogs as a basis for this study. The first is a catalog of all magnetopause and bow shock crossings by the Cassini spacecraft during its 13‐year orbital tour of Saturn (Jackman et al., 2019). This list is established using the magnetic field measurements (Dougherty et al., 2004). It enables us to unambiguously select times when Cassini was in the magnetosheath and to focus on those for the observation of the NB. The second is the catalog of 5 kHz NB from S. Wu et al. (2021). The global distribution of the 5 kHz L‐O mode NB (the 5 kHz Z‐mode NB is not plotted) in the meridional plane is shown in Figure 1 panel (a). The gap region in the equatorial area (the black dashed circles) is due to the blocking of the 5 kHz NB by the Enceladus plasma torus (the rough location of the Enceladus plasma torus is 2–21 Rs in the equatorial region; Persoon et al., 2013). Because the L‐O mode cannot pass through the dense part of the plasma torus, the original L‐O mode NB generated interior to the plasma torus would propagate to high latitudes as indicated by the black arrows in panel (a). But how did these NB bypass the plasma torus and reach the low latitude region outside the plasma torus?

The region in Figure 1 panel (b) enclosed by the iso‐surfaces of *f*_uh_ and *f*_L = 0_ marks the theoretical trapping region of the 5 kHz Z‐mode NB. It consists of the pink region around Saturn and a “channel” which goes around the Enceladus plasma torus. It has been shown by S. Wu et al. (2021) that Z‐mode NB were observed around Saturn and close to the inner edge of the plasma torus (pink region), but not in the middle and outer parts of the channel. This could be due to the fact that the channel is a dispersive medium, and in principle each frequency has its own channel. It is therefore unlikely that a Z‐mode NB emission as a whole can propagate to the outer parts of the channel and mode convert to L‐O mode there. Furthermore, a possible reflection of the Z‐mode can only happen at *f*_L = 0_, but not at *f*_uh_ as the emissions get absorbed near the upper hybrid resonance. Consequently, the mode conversion from Z‐mode to L‐O mode NB should most likely happen at the density gradients on the inner edge of the plasma torus, which again poses the question of how NB emissions can bypass the plasma torus.

## 3 The "Missing" 5 kHz NB Emissions

**Figure 2:**
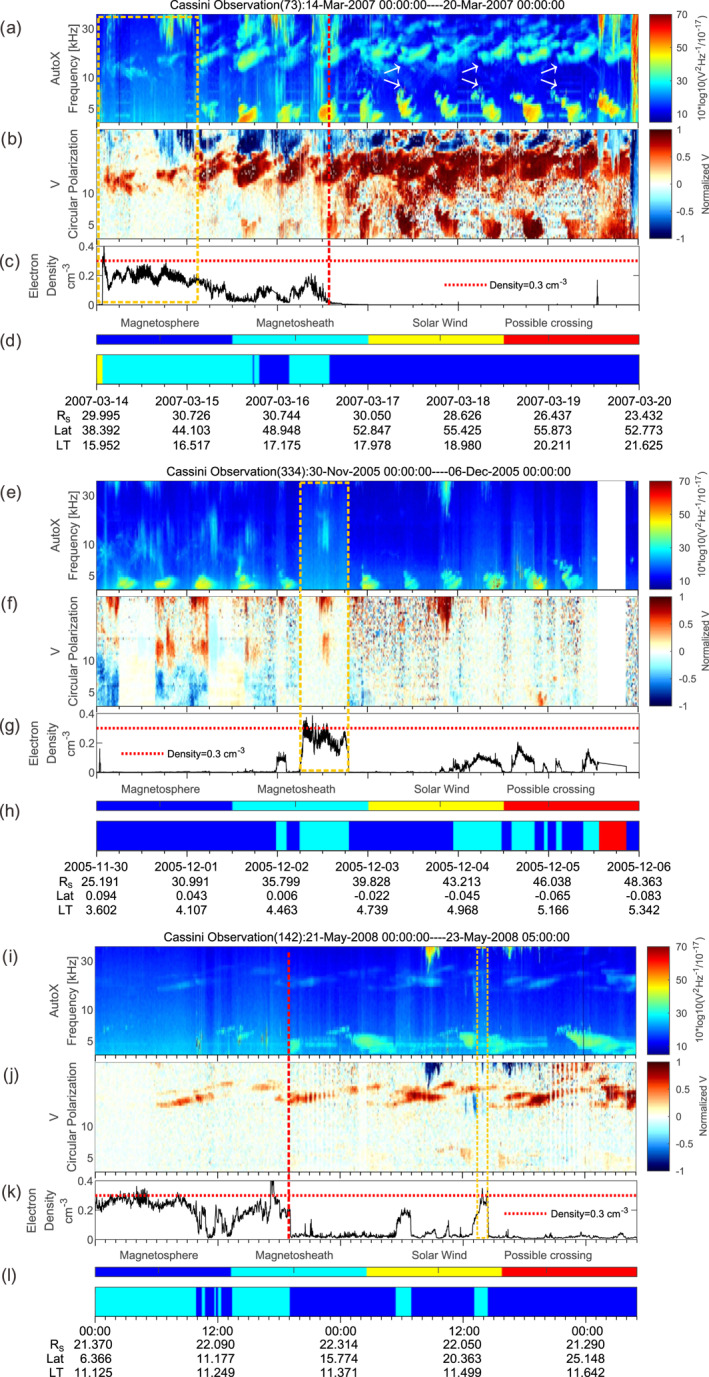
Panels (a)–(d). Wave electric field spectrogram; spectrogram of the normalized Stokes parameter, V for circular polarization (1: left‐hand circular polarization, −1: right‐hand); electron density; the location of Cassini from Jackman et al. (2019). The vertical red dashed line marks the location of the magnetopause. The white arrows indicate the 5 and 20 kHz NB. Panels (e)–(h), (i)–(l) show the other two cases. The vertical yellow dashed boxes mark the “missing” NB.

Three observations are given in Figure 2 to illustrate the "missing" 5 kHz NB phenomenon. The electric field data in panel (a), (e), and (i) are the autocorrelation values measured by the Radio and Plasma Wave Science (RPWS) antenna onboard Cassini (Gurnett et al., 2004). The signals near 5 and 20 kHz modulated at a period near Saturn's rotation (∼10.6 hr) are the NB emissions (indicated by the white arrows in panel (a) (Ye, Gurnett, et al., 2010). Sometimes the SKR extends down to 20–30 kHz from the top of the spectrum as shown in panels (a), (e) and (i).

The Stokes parameters V (in panels (b), (f), (j)) are obtained by assuming the waves originated from the center of Saturn (detailed in Cecconi & Zarka. (2005) and Lamy et al. (2011)). The L‐O mode NB emissions would show left‐hand circular polarization (Stokes parameter V ∼ = 1) in the northern hemisphere and right‐hand circular polarization (V ∼ = −1) in the southern hemisphere due to the variation of the angle between the wave vector and the background magnetic field (Ye, Menietti, et al., 2010). It is just the other way around for the R‐X mode SKR. As shown in panel (b), (f), both the 5 and 20 kHz NB are circularly polarized. The 5 kHz NB are often unpolarized as shown in panels (f) and (j) (S. Wu et al., 2021), which could be due to the reflection and refraction by the magnetosheath. The electron density (panels (c), (g), (k)) are obtained from the electron moments data generated from the Cassini Plasma Spectrometer (CAPS) electron spectrometer (Young et al., 2004). The 5 kHz NB observed in these three cases are L‐O mode emissions, and they cannot propagate in regions with plasma frequencies (*f*_pe_) higher than the wave frequency. A *f*_pe_ of 5 kHz corresponds to an electron number density of 0.3 cm^−3^. The electron density in the magnetosheath is higher than in the magnetosphere, where it is close to zero, and it is mostly below 0.3 cm^−3^ and above this level only occasionally.

The yellow dashed boxes in panels  (a), (e) and (i) mark the "missing" 5 kHz NB. In panel (a), Cassini was first in the high density region in the magnetosheath. No 5 kHz NB was observed at this time as indicated by the red box. When Cassini entered the magnetosphere, the 5 kHz NB appeared. In this example, the time within the red box has a density close to but below 0.3 cm^−3^, so the “missing” 5 kHz NB was possibly refracted to other directions and thus could not reach Cassini. Near 3–15 4:48 and 3–16 6:00, the 5 kHz NB signals with diffuse boundaries could be attributed to the attenuation by the magnetosheath. In comparison, the 20 kHz NB can propagate across the denser magnetosheath into the solar wind. It is worth noting that the 20 kHz NB seemed to be weaker at the times of the “missing” 5 kHz NB, probably due to refraction by the magnetosheath. The maximum frequency that the magnetosheath can reflect may be influenced by the geometric effect and change as *f*_wave_ = *f*_pe_/cos (*α*; *α* is the incident angle; Ye et al., 2012). For a density structure with a plasma frequency f_pe_ equal to 5 kHz, we presume the incident angle is 60°. Then the wave frequency would need to be at least 10 kHz for this oblique wave to go through. If the wave frequency is equal to 5 kHz and with the same incident angle, the plasma frequency *f*_pe_ in the structure only needs to be above 2.5 kHz (corresponding to 0.0775 cm^−3^) for reflection to occur. Therefore, the absolute value of the electron density needed for the magnetosheath to reflect NB could be even lower.

Studies showed that the 5 kHz NB are rotationally modulated (Wang et al., 2010; Ye et al., 2016), and the periodicity matched the periods of the so‐called type 2 plasma injection events (Azari et al., 2018; Mitchell et al., 2015; Wing et al., 2020). If the 5 kHz NB is generated by a magnetospheric process, for example, plasma injection, then it is possible that the 5 kHz NB would not be generated during periods of no injection. However, the “missing” emission in panel (e) is observed in the middle of a series of 5 kHz NB emissions and the location of the Cassini spacecraft during the 6 days did not vary much. The disappearance of the NB is almost coincident with the electron density peak surpassing the density of 0.3 cm^−3^. The "missing" signal should not be "no excitation" or a geometry effect of the observation location. This case can again be explained by NB not being able to propagate in the magnetosheath due to the high plasma density.

There was a second excursion of Cassini through the magnetosheath after 12‐04 00:00 UT in Figure 2 panel (e). However, no NB is missing because the electron density is significantly lower and less than 0.15 cm^−3^, which was evidently not high enough for reflection to occur. The NB near 12‐4, 19:11 UT, which has the opposite polarization sense (*V* > 0), could possibly be due to a reflection and will be discussed in Section 4. In panel (i), the evidence is even more obvious since the “missing” NB in this case is just a part of the NB signal. The NB signal disappeared after a sudden crossing of the dense magnetosheath, and the signal was received immediately after exiting the magnetosheath. Near 5–21 21:12 UT in panel (i), a sudden vertical cut‐off of NB at the magnetopause is observed and this will be discussed in the next section in combination with two other cases.

## 4 The Sudden Cut‐Off and Reflection at the Magnetopause

**Figure 3:**
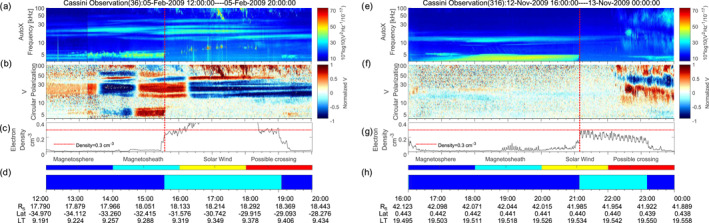
The sudden cut‐off cases of 5 kHz NB. Similar format as Figure 2.

Three cases of sudden cut‐offs are shown in Figure 2 panels (i)–(l) and Figure 3. The vertical red dashed lines mark the position of the magnetopause. In all three cases, the electron density jumped at the magnetopause from a small value in the magnetosphere to near 0.3 cm^−3^ in the magnetosheath. At the same time, the 5 kHz NB stop at the magnetopause.

The case in the left panels of Figure 3 took place at medium southern latitudes. In the magnetosphere, the 5 kHz NB is a highly polarized emission with a high circular polarization (panel (b)). This suggests that the emission was directly beamed from the source to medium latitudes and experiences a full reflection at the magnetopause, as in the magnetosheath Cassini cannot see any trace of the emission in the intensity or the polarization plots. This is somewhat different for the case in the right panels of Figure 3, which took place around equatorial latitudes. Here the polarization plots indicate that the incident wave is practically unpolarized, and it might have already undergone reflections at other places.

The NB disappeared in the magnetosheath and re‐appeared in the magnetosphere for both cases in Figure 3. This provided us a chance to verify the local *f*_pe_ at the magnetopause by using the NB cut‐off frequency in the spectrum. The bursty emissions near 10 kHz in the magnetosheath in panel (a) are Langmuir waves which could also be used to derive the local plasma density.

**Figure 4:**
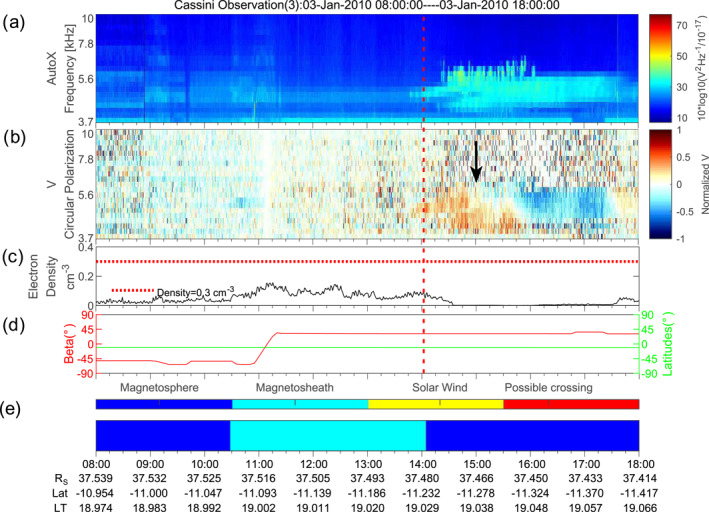
The reflected 5 kHz NB. The black arrow in panel  (b) marks the reversal of the circular polarization. Panel  (d) gives the calculated angle (Beta) between the incoming wave direction and the antenna plane.

We show a 5 kHz NB case possibly reflected near the magnetosheath in Figure 4. Cassini was located in the southern hemisphere at this time. The L‐O mode would show a circular polarization degree close to −1 (in blue) due to the angle between the wave vector and the background magnetic field exceeding 90°. However, as shown in panel  (b) and marked by the black arrow, the red part of NB would correspond to an R‐X mode wave. The Stokes parameters are obtained by assuming that the wave vector is pointing from the center of Saturn to the spacecraft. Sometimes Cassini was rolling, which will cause a flip of the polarization sense in the data (e.g., in panel (b) of Figure 3). During this time, the calculated angle beta (defined as the angle between the direction of Saturn and the normal direction of the antenna plane as described in Ye et al. (2009)) in panel  (d) was stable near 30°, that is, Cassini was not rolling. Therefore the red part (corresponding to left‐hand polarized) will be either R‐X mode emissions or L‐O mode emissions incident from the other side of the antenna plane, which is directed toward the magnetosheath. The blue part is probably L‐O mode emission coming directly from Saturn, that is, in the magnetosphere Cassini probably detected waves coming directly from Saturn and those reflected by the magnetosheath.

In the magnetosphere near the magnetopause NB should propagate in L‐O mode. One might also interpret this as mode conversion from the L‐O mode to Z‐mode at a density gradient near the magnetopause. Then the “flip line” of polarization in the spectrogram can be explained as the local *f*_pe_. The Z‐mode would correspond to the left side signal which is below the *f*_pe_. However, the Z‐mode below *f*_pe_ has the same sense of polarization as the L‐O mode above *f*_pe_, which contradicts the observations. Therefore, the reversal of the polarization sense should be attributed to the reflection of NB near the magnetosheath. Similar features may have been detected as well for the examples of Figure 3, but Cassini never seemed to have crossed the beam of reflected rays due to a large incidence angle at the magnetopause. The reflected NB in Figure 4 (red part, left‐hand polarized) signal is incident on the antenna plane from the other side relative to the out‐going NB (blue part, right‐hand polarized in antenna frame, left‐hand polarized with respect to magnetic field). We note that the plasma density in the magnetosheath only goes up to ∼0.15 cm^−3^, corresponding to 3.5 kHz. However, an angle of incidence of *α* = 60° would suffice to provide reflection for all frequencies below 7 kHz. The change in polarization is clearly a function of frequency. This might be explained by a reflection of different frequencies in different plasma layers, and a scattering of reflected rays (similar to scattering of white light into rainbow colors when going through a prism).

## 5 The Variation of Bandwidth

The reflection‐by‐sheath mechanism could also explain the variation of the upper frequency limit of the 5 kHz NB.

**Figure 5:**
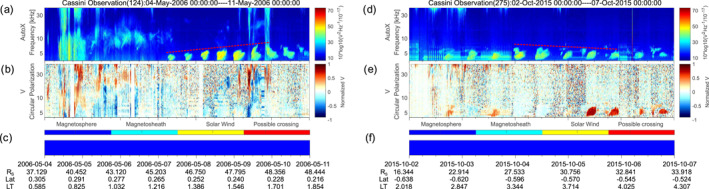
The drift of the upper frequency limit of 5 kHz NB. Similar format as Figure 2. The red lines in panels  (a) and (d) mark the trend of up and down drift of the upper frequency limit of NB.

When the density in the magnetosheath increases, the upper frequency limit of NB reflected by the magnetosheath will also increase due to the increased plasma frequency in the magnetosheath. As marked by the red lines in Figure 5, panels  (a) and (d), the upper frequency limits of the 5 kHz NB drift to higher and lower frequencies. These two cases are observed at low latitudes outside the plasma torus after reflection of 5 kHz NB by the magnetosheath. Hence, the left panels would correspond to a situation with increasing plasma density in the magnetosheath (at the reflection point) due to compression by the solar wind. Similarly, the right panels correspond to a decreasing plasma density and relaxation of the magnetosphere. The left case has been reported in detail by Jackman et al. (2010) as a typical compression event. Their study suggested a main compression during the earlier days roughly from May 4 to May 7^,^ and a relaxation phase from May 7 to May 9, exactly during the frequency drift period of the NB as shown in panel (a). This suggests that the density increase at the reflection point does not vary synchronously with large‐scale solar wind conditions but with some time delay. It is possibly related to the response times of density structures inside the magnetosheath to changes in the solar wind, like Kelvin–Helmholtz instability vortices, which are a topic of ongoing research (Burkholder et al., 2020; Masters et al., 2010).

## 6 Discussion

Ye, Menietti, et al. (2010) showed evidence that Z‐mode NB is the source of L‐O mode NB. This earlier study suggested that Z‐mode NB could propagate along the “Channel” and mode convert to the L‐O mode at the outer edge of the plasma torus. However, the statistical study of S. Wu et al. (2021) showed practically no observation of the 5 kHz Z‐mode NB inside the narrow channel region around the torus. The channel is actually a dispersive medium and each frequency has its own channel. We speculate that the Z‐mode could not propagate inside the narrow “Channel” but are only trapped interior to the plasma torus as marked by the pink region in Figure 1 panel (b). A ray‐tracing study at Earth suggested the Z‐mode could be bent to propagate parallel to the local *f*_ce_ iso‐surface when the electron density gradient is perpendicular to the magnetic field (Gurnett et al., 1983). A ray‐tracing study using the Saturnian parameters will be carried out in the future to cover this topic.

Cassini spent roughly 9.08 years, 2.15 years, and 1.72 years in the magnetosphere, the magnetosheath, and the solar wind, respectively. The observed NB durations are 0.91 years (1975 cases), 0.063 years (180 cases), and 0.001 years (4 cases) in the corresponding regions. The occurrence rate of NB in the magnetosphere is approximately 10% (∼0.91 years/9.08 years). The rough occurrence rates are 2.9% and 0.06% in the magnetosheath and solar wind, respectively. The differences in the occurrence rates may imply that most of the 5 kHz NB are “trapped” in the magnetosphere by the dense magnetosheath. Besides, based on the derived electron density data in Saturn's magnetosheath (Thomsen et al., 2018), if all NB arrived at the magnetopause with an average angle of 45°, the required electron number density for the 5 kHz NB to be reflected would be just 0.155 cm^−3^. This value would correspond to approximately 37% of the time when Cassini was inside the magnetosheath (73% for incidence angle *α* = 60°).

This work discussed several instances in which 5 kHz NB were reflected or refracted by the magnetosheath. The reflection‐by‐sheath mechanism can explain the low latitude cases observed outside the plasma torus, the polarization features and the drift of the upper frequency limit of the 5 kHz NB. This new insight of the 5 kHz NB make the reflected NB a possibly useful tool to sense the magnetosheath remotely.

## Data Availability

The Cassini MAG data and CAPS data were downloaded from the Planetary Data System at (MAG: https://pds-ppi.igpp.ucla.edu/search/view/?f=yes&id=pds://PPI/CO-E_SW_J_S-MAG-4-SUMM-1MINAVG-V2.0/DATA; CAPS: https://pds-ppi.igpp.ucla.edu/search/view/?f=yes&id=pds://PPI/CO-E_J_S_SW-CAPS-5-DDR-ELEMOMENTS-V1.0/DATA). The Cassini RPWS data used in this work were downloaded from the LESIA/Kronos collection with n2 level data (Cecconi et al., 2017a) and n3d data (Cecconi et al., 2017b; goniopolarimetric data obtained using the method Cecconi & Zarka. 2005). The boundary crossing list can be obtained from Jackman et al. (2019) through the website (https://agupubs.onlinelibrary.wiley.com/doi/abs/10.1029/2019JA026628). The NB list can be obtained from the supplementary material at (https://iopscience.iop.org/article/10.3847/1538-4357/ac0af1#apjac0af1t1) and also from the data repository at LESIA via a DOI link (S. Y. Wu et al., 2021).
